# Evaluation of rhizosphere, rhizoplane and phyllosphere bacteria and fungi isolated from rice in Kenya for plant growth promoters

**DOI:** 10.1186/2193-1801-2-606

**Published:** 2013-11-13

**Authors:** Mwashasha Rashid Mwajita, Hunja Murage, Akio Tani, Esther M Kahangi

**Affiliations:** Kenya Agricultural Research Institute, PO Box 57811-00200, Nairobi, Kenya; Jomo Kenyatta University of Agriculture and Technology, PO Box 62000-0020, Nairobi, Kenya; Institute of Plant Science and Resources Okayama University, 2-20-1 Chuo, Kurashiki, Okayama, 710-0046 Japan

**Keywords:** Micro-organisms, Phosphate solubilization, Nitrogen fixation, IAA production

## Abstract

Rice (*Oryza sativa* L.) is the most important staple food crop in many developing countries, and is ranked third in Kenya after maize and wheat. Continuous cropping without replenishing soil nutrients is a major problem in Kenya resulting to declining soil fertility. The use of chemical fertilizers to avert the problem of low soil fertility is currently limited due to rising costs and environmental concerns. Many soil micro-organisms are able to solubilize the unavailable phosphorus, increase uptake of nitrogen and also synthesize growth promoting hormones including auxin. The aim of this study was to isolate and characterize phyllosphere, rhizoplane and rhizosphere micro-organisms from Kenyan rice with growth promoting habits. In this study whole plant rice samples were collected from different rice growing regions of Kenya. 76.2%, over 80% and 38.5% of the bacterial isolates were positive for phosphate solubilization, nitrogenase activity and IAA production whereas 17.5% and 5% of the fungal isolates were positive for phosphate solubilization and IAA production respectively. Hence these micro-organisms have potential for utilization as bio-fertilizers in rice production.

## Introduction

Rice (*Oryza sativa* L.), which is one of the most important food crops in the world, nourishing approximately 50% of the population and directly providing 20% of human calorie intake (Zeigler and Barclay 2008). It is the most important staple food crop in many developing countries, and is ranked third in Kenya after maize and wheat. In Kenya, the annual rice consumption is increasing at the rate of 12% compared to wheat (4%) and maize (1%) (GoK 2009).

Declining soil fertility as a result of continuous cropping without replenishing soil nutrients is a major problem in Kenya. Microorganisms are utilized in agriculture for various purposes; as important components of organic amendments and composts, as inoculants for biological nitrogen fixation, phosphorous solubilization and indole acetic acid (IAA), to improve crop quality and yields (Higa 1994).

The rice plant represents a habitat for diverse microorganisms, those that colonize the aerial parts (phyllosphere), the root surface (rhizoplane) as well as the zone around the root (rhizosphere) (Knief et al. 2011). The phyllosphere comprises the aerial parts of plants and is dominated by the leaves. Most studies on the identity of organisms in the phyllosphere have focused on bacteria and, to a lesser extent, fungi (Vorholt 2012). Potential beneficial interactions of phyllosphere bacteria with rice plants, such as plant growth promotion by bacterial nitrogen fixation or plant hormone production have been studied (Knief et al. 2011). Rhizoplane is the root surface zone where microorganisms attach themselves using surface structures such as flagella, fimbriae or cell surface polysaccharides. The boundary between rhizoplane and rhizosphere is very thin and therefore this habitat is largely considered as a continuum. (Johri et al. 2003). The rhizosphere is a thin layer of soil immediately surrounding plant roots. This is an extremely important and active area for root activity and metabolism. A large number of microorganisms such as bacteria, fungi, protozoa and algae coexist in the rhizosphere. Bacteria are the most abundant among them (Saharan and Nehra 2011).

One of the most common strategies to increase agricultural production is through the improvement of soil fertility. Nitrogen (N) and phosphorus (P) are the two most limiting nutrients in soil. Indole acetic acid is an essential natural growth promoter that extensively affects plant growth and development. Cereal plants require large amounts of mineral nutrients including N for their growth, development and grain production (Baset Mia and Shamsuddin 2010). Nitrogen fixation and plant growth enhancement by microorganisms might be important factors for achieving a sustainable agriculture. Micro-organisms with the ability to reduce and derive appreciable amounts of nitrogen from the atmospheric reservoir and enrich the soil include bacteria and archaea (Saharan and Nehra 2011). Environmental problems caused by the use of inorganic fertilizers may therefore be reduced through use of microorganisms which enhance N uptake by plants.

The second most mineral nutrient limiting the growth of crops is P. Phosphorus is an essential macronutrient for plant growth and development. Due to P fixation and precipitation which occur in soil, the concentration of soluble P in soil is usually very low (Fankem et al. 2008) especially in sub Saharan Africa. Soil microorganisms are known to be effective in releasing P from organic pools of total soil P by mineralization and from inorganic complexes through solubilization. Through their metabolic activities, by exudating organic acids which directly dissolve the rock phosphate, or chelate calcium ions, many soil microorganisms are able to solubilize the unavailable P and release it to the solution (Abd-Alla 1994).

Auxin is the first phytohormone to be identified among plant hormones which plays an important role in root system development and plants yield. Indole acetic acid is the common natural auxin that shows all auxin activity and extensively affects pants physiology (Etesami et al. 2009). Reports have indicated the ability of many bacterial microorganisms to produce phytohormones that can enhance the plant root contact surface with soil and subsequently the increase of nutrient uptake via root elongation. Due to this ability, microorganism inoculants can be used as a substitute for chemical fertilizers in partially fertile soils and/or at least as a supplement for chemical fertilizers in infertile soils.

Knowledge on which microorganisms can biologically fix nitrogen, solubilize phosphorus and induce substances like IAA that can contribute to the improvement of rice growth hence depending less on chemical fertilizers, is crucial for sustainable rice cultivation. Therefore, the present study was undertaken to screen the rhizosphere, rhizoplane and phyllosphere bacteria and fungi isolated from rice growing regions of Kenya for their physiological characteristics, including P-solubilization, N-fixation and IAA production.

## Results

### Isolation of micro-organisms

A total of 130 pure bacterial and 120 pure fungal isolates were obtained from the rhizosphere, rhizoplane and phyllosphere samples from the three sites. Out of the total 250 pure isolates, Coast had the highest number (38.8%), followed by Mwea with 34.8% then Western with 26.4% irrespective of whether they are bacterial or fungal, from the rhizosphere, rhizoplane or phyllosphere samples (Table 1).

**Table 1:** **Isolates from various sources**

Origin/Site	Number of isolates					
	Rhizoplane		Rhizosphere		Phyllosphere	
	Bacteria	Fungi	Bacteria	Fungi	Bacteria	Fungi %
**Mwea**	29	31	5	6	9	7 34.8
**Western**	6	16	4	4	19	17 26.4
**Coast**	27	10	21	14	10	15 38.8
**Total**	62	57	30	24	38	39 100.0

### Characterization of bacterial isolates

The morphological characteristics of the bacterial isolates varied widely as shown in (Table 2). All the isolates produced round shaped colonies, the elevation was either raised or convex, had smooth or undulate margin with the colour ranging from white to brown. Microscopic observations were performed to investigate the some characteristics of the isolates such as cell shape, Gram reaction and motility (Table 3). All the isolates were motile, the cell shape was mostly rod and majority were Gram positive in reaction.

**Table 2:** **Morphological characteristics of bacterial isolates**

No. of isolates	Shape	Elevation	Margin	Colour
19	Round	Raised/convex	Smooth/undulate	Whitish
83	Round	Raised/convex	Smooth/undulate	Off whitish
27	Round	Raised/convex	Smooth/undulate	Yellowish
1	Round	Raised/convex	Smooth/undulate	Brownish

**Table 3:** **Microscopic observation of bacterial isolates**

No. of isolates	Cell shape	Motility	Gram reaction	Genus
82	Rod/cocci	Motile	Positive	*Bacillus* spp.
8	Rod	Motile	Negative/positive	*Pseudomonas* spp.
8	Rod/cocci	Motile	Positive	*Enterobacter* spp.
6	Rod	Motile	Positive	*Lysinbacillus* spp.
6	Rod/cocci	Motile	Positive	*Staphylococcus* spp.
3	Rod	Motile	Negative	*Micrococcus* spp.
3	Rod	Motile	Positive/negative	*Streptomyces* spp.
3	Rod/cocci	Motile	Negative/positive	*Brevundomonas* spp.
2	Rod	Motile	Positive	*Acinetobacter* spp.
2	Rod	Motile	Positive	*Serratia* spp.
2	Rod	Motile	Positive	*Vagococcus* spp.
2	Rod	Motile	Negative	*Exiguobacterium* spp.
1	Rod	Motile	Positive	*Alcaligenes* spp.
1	Rod	Motile	Positive	*Brevibacillus* spp.
1	Rod	Motile	Positive	*Advenella* spp.

### Characterization of fungal isolates

The fungal isolates were morphologically characterized according to the colony surface, reverse and periphery colour. The isolates varied widely in colours for all the attributes. With the assistance of illustrations by Barnett and Hunter (1987), the isolates were microscopically identified at the Genus level into eight groups (Table 4).

**Table 4:** **Colony characteristics of fungal isolates**

No. of isolates	Surface colour	Reverse colour	Periphery colour	Genus
47	Dark green	Yellow/brown	Whitish	*Penicillium* spp.
29	Sulphur yellow	Creamish	Whitish	*Aspergillus* spp.
24	Grey	Yellow	Creamish	*Trichoderma* spp.
9	Grey/Green	Yellow	Whitish/Creamish	*Eupeniccilium* spp.
6	Dark green	Creamish	Dark green	*Isaria* spp.
3	Whitish	Creamish	Whitish	*Leptosphaerulina* spp.
1	Fiesta green	Creamish	Fiesta green	*Hypocrea* spp.
1	Pink	Red	Pink	*Fusarium* spp.

### Nitrogen fixation ability

**Figure 1:**
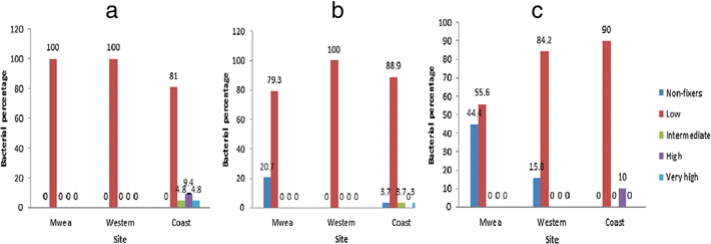
**(a–c) Acetylene reduction activity by bacterial isolates.** Non-fixers = 0, Low = 0.1-20, Intermediate = 21–40, High = 41–60, Very high >60 nmol of C2H4/tube/12 h.

N_2_-fixing ability was evaluated and the isolates grouped into five groups; Non-fixers (0 nmol of C_2_H_4_/tube/12 h), Low (0.1–20 nmol of C_2_H_4_/tube/12 h), Intermediate (21–40 nmol of C_2_H_4_/tube/12h), High (41–60 nmol of C_2_H_4_/tube/12 h) and Very high (>60 nmol of C_2_H_4_/tube/12 h). The capability of rhizosphere, phyllosphere and rhizoplane bacterial isolates from Mwea, Western and Coastal regions to fix nitrogen was examined based on isolates ability to reduce acetylene (C_2_H_2_) to ethylene (C_2_H_4_). The reduction of C_2_H_2_ to C_2_H_4_ is widely used to assess nitrogenase activity in natural isolates, a method commonly known as Acetylene reduction activity (ARA). All the (30) rhizospheric bacterial isolates from the three sites showed (ARA) (Figure 1a) albeit at different levels. Most of these isolates were low fixers with only Coastal region isolates which had fixing ability in the intermediate, high, and very high groups.

Over 70% rhizoplane bacteria from the three sites were able to fix nitrogen (Figure 1b). At low levels, over 50% of the phyllosphere bacteria from the three sites were able to fix nitrogen (Figure 1c).

### Phosphate solubilization ability

**Figure 2:**
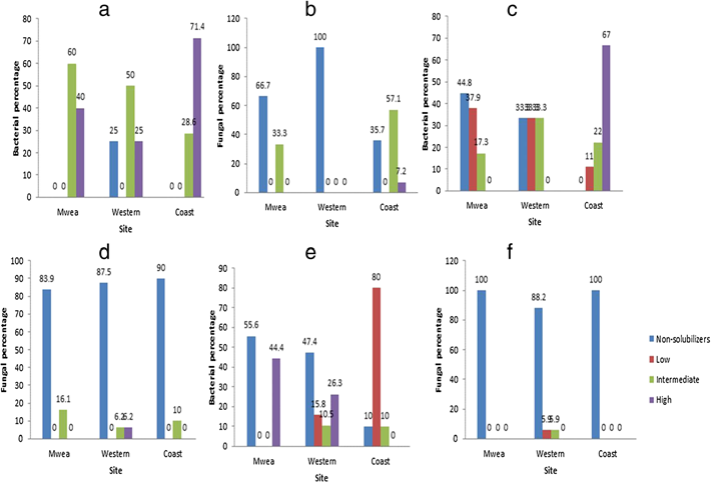
**(a–f) P-solubilization by bacterial and fungal isolates.** Non-solubilizers = 0 mm, Low = 1–10 mm, Intermediate = 11–20 mm, High ≥ 21 mm; Halo size.

Ninety nine out of 130 bacterial isolates were able to solubilize phosphates while only 21 out of 120 fungal isolates produced halos both in NBRIP growth medium with and without bromophenol blue. Based on the results, the isolates were classified into four groups depending on the halo size; Non-solubilizers (0 mm), Low (1–10 mm), Intermediate (11–20 mm) and High (≥ 21 mm) solubilizers. Phosphate solubilization was most frequently encountered in the rhizospheric bacteria where Mwea and Coastal regions had 100% solubilization ability distributed within intermediate and high (Figure 2a). Most of the rhizospheric fungi were non-solubilizers (Figure 2b) with Western region having 100% nonsolubilization ability. Rhizospheric fungi from Western region were non-solubilizers (Figure 2b) while 64.3% and 33.3% isolates from Coast and Mwea, respectively, were solubilizers. All the 27 rhizoplane bacterial isolates from the Coastal region were able to solubilize phosphates as opposed to those of Mwea and Western region (Figure 2c). Ability of the rhizoplane bacterial isolates from the Coastal region to solubilize phosphates was 100% (Figure 2c) compared to Western (66.6%) and Mwea (55.2%). More than 80% of soil fungi from all the 3 regions were non-phosphate solubilizers while Mwea had the highest percentage (16.1%) of soil fungi which could solubilize phosphates (Figure 2d). Western region had its phyllosphere bacterial isolates distributed within all the 4 groups, with the majority (47.4%) being non-solubilizers (Figure 2e) as opposed to Mwea and Coastal regions. All the phyllosphere fungal isolates from Mwea and Coastal regions as well as over 80% from Western were non-solubilizers (Figure 2f).

### IAA production ability

**Figure 3:**
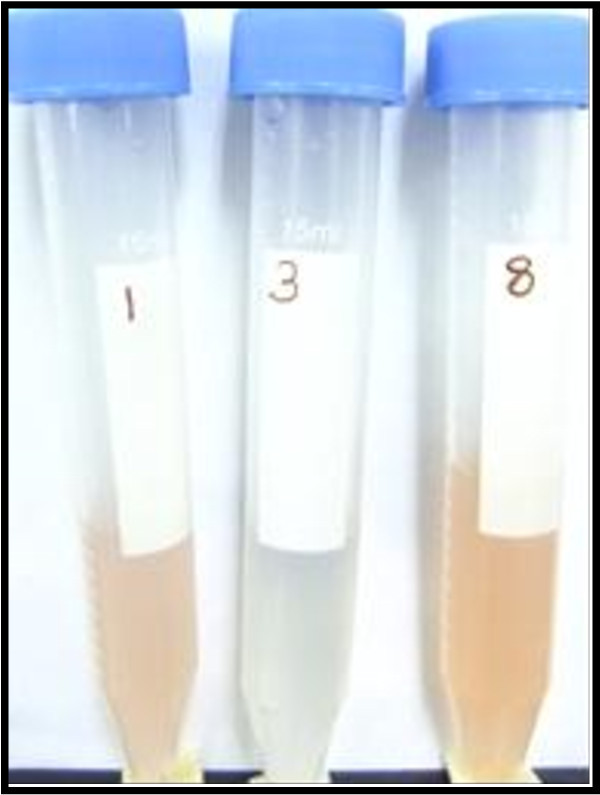
**Isolates showing differences in ability for IAA production.**

**Figure 4:**
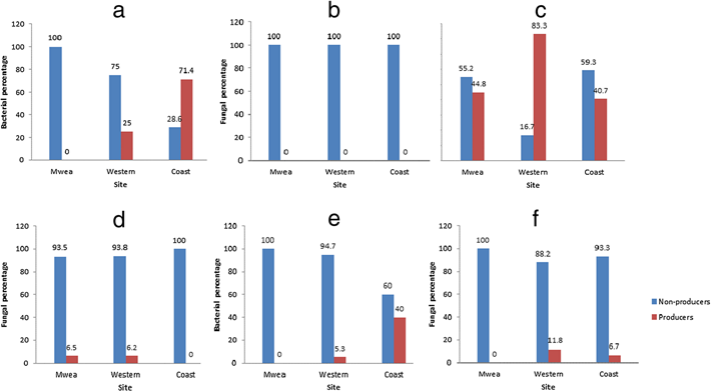
**(a–f) IAA production by bacterial and fungal isolates.** IAA production was detected by the development of pink colour after the addition of salkowski reagent to the cultures

The ability of the microorganisms to produce IAA was detected by the development of pink colour after the addition of salkowski reagent to the cultures as shown in Figure 3. All the Mwea rhizospheric bacterial isolates and 75% of Western isolates were unable to produce IAA whereas over 70% (Figure 4a) of those from the Coastal region were IAA producers. Hundred percent of the fungal isolates (Figure 4b) from the 3 regions were unable to produce IAA. The rhizoplane of the 3 regions contained both the IAA bacterial producers and non-producers at varying intensities (Figure 4c). Western region had more (83.3%) IAA producers than non-producers (16.7%) as opposed to the other 2 regions. Fungal non-producers dominated the rhizoplane of the 3 regions (over 90%) with Coastal region having no IAA producer (Figure 4d). The composition of phyllosphere bacterial isolates was very minimal with the exception of Coastal region where the IAA producers were 40% (Figure 4e). Over 80% of the phyllosphere fungal isolates were non-producers with Mwea region having 100% IAA non-producers (Figure 4f).

## Discussion

It is evident from these results that the above- and below-ground parts of the rice plants are a habitat for diverse microorganisms (Table 1). These findings are consistent with those of Prasanna et al. (2011) who reported that rice fields represent unique aqua-terrestrial ecosystems with tremendous diversity of soil microbes including bacteria and fungi amongst others. The bulk soil (rhizoplane) had the highest number of micro-organisms as opposed to the rhizosphere and phyllosphere. This is because the phyllosphere is a short-lived environment, as opposed to the rhizoplane which comprises the area in the soil around plant roots. The number of bacterial isolates from the rhizoplane and rhizoshere were more than that of the fungal isolates. This is in agreement with Saharan and Nehra (2011) who reported that bacteria are the most abundant microorganisms in the rhizosphere.

The reduction of C_2_H_2_ to C_2_H_4_ is equivalent to the measure of the total amount of nitrogen that an organism has fixed. The usefulness of ARA methodology in screening plants and microorganisms for presence of nitrogenase activity is beyond doubt (Zafar et al. 1986). There was a wide range of variation in nitrogenase activity among the 130 isolates tested in accordance with the regions and samples. Knief et al. (2011) reported that nitrogen fixing bacteria are present in the rhizosphere, rhizoplane as well as the phyllosphere. One of the important components of the nitrogen cycle in a range of ecosystems, is nitrogen fixation associated with roots of grasses. The rhizosphere is known to be a biologically active zone that contains soil-borne microbes where the biological, chemical and physical characteristics influence the roots. A large number of microorganisms such as bacteria and fungi coexist in the rhizosphere and bacteria are the most abundant among them. Zafar et al. (1986) reported high acetylene-reducing activities associated with soil (rhizoplane) and roots of kallar grass. Despite the ability of rhizoplane isolates to fix nitrogen in the 3 regions, most (> 70%) of them were low fixers (Figure 1b). There are reports on characterization of a number of bacterial isolates from the rice phyllosphere and potential beneficial interactions of phyllosphere bacteria with rice plants, such as plant growth promotion, by bacterial nitrogen fixation. Thus in this study most (> 50%) of the phyllosphere bacterial isolates from the 3 regions also showed some ARA as low fixers (Figure 1c). However, most of the non-fixer microbes in the 3 regions were detected in the phyllosphere where Mwea and Western regions had 44.4% and 15.8% respectively as opposed to the rhizoplane where Mwea and Coastal regions had 20.7% and 3.7% respectively and the rhizosphere which had 0% non-fixers. The results indicate that the rhizosphere and the rhizoplane microbes have better ARA than those at the phyllosphere. Knief et al. (2011) detected genes encoding dinitrogen reductase and dinitrogenase in the rhizosphere and the phyllosphere metagenome with a few peptides of dinitrogenase reductase identified in the rhizoplane. From their observation, the nitrogen fixing bacteria are present in the rhizosphere, rhizoplane as well as the phyllosphere.

Soil microorganisms are known to be effective in releasing P from inorganic complexes through solubilization. The ability of microorganisms to solubilize phosphates is detected by production of clear zone/halo around colonies/structures on media containing insoluble mineral phosphate. According to Rodríguez and Fraga (1999) the halos formed around the colonies is as a result of pH drop produced by the release of organic acids, which are responsible for phosphate solubilization. In this study most (99) of the bacterial isolates from the 3 regions and 21 fungal isolates were able to solubilize phosphate. These results indicate that rhizospheric bacteria have the ability to solubilize precipitated phosphates as reported by Verma et al. (2001). According to Johri et al. (2003) in addition to rhizobacteria, several fungi such as species of *Aspergillus* can efficiently solubilize P. Many researchers have reported that almost all phosphate solubilizing microorganisms when multiplied on a simple “C” source, produce gluconic, glycolic and 2-ketogluconic acids among others in the medium which are responsible for P solubilization. Over 50% of the phyllosphere bacterial isolates from Western and Coastal regions were able to solubilize phosphates (Figure 2e) whereas over 80% of the phyllosphere fungal isolates from the 3 regions were non-solubilizers (Figure 2f). Even though the fungal isolates were able to solubilize phosphates to some extent, their ability was not comparable to that of the bacterial isolates. In general the bacterial isolates from the rhizosphere and rhizoplane were more efficient in solubilizing phosphate than the fungal isolates. These results indicate that there are considerable populations of phosphate-solubilizing bacteria in soil and in plant rhizosphere as indicated by Alexander (1977) who reported that there are considerable populations of phosphate-solubilizing bacteria in soil and in plant rhizosphere.

Out of the many phytohormones, IAA is generally considered to be the most important native auxin. According to Leinhos (1994) bacterial biosynthesis of IAA is known in many rhizobacteria and it is believed that approximately 80% of rhizospheric bacteria can secrete IAA. However, Joseph et al. (2007) reported that the ability of bacteria to produce IAA in the rhizosphere depends on the availability of precursors and uptake of microbial IAA by plant. In the present study, over 70% (Figure 4a) of rhizospheric bacterial isolates from the Coastal region were IAA producers whereas 75% of Western isolates and all the Mwea isolates were unable to produce IAA. Compared to the rhizospheric bacterial isolates, the rhizosplane bacteria had a greater percentage of IAA producers (Figure 4c). These results differed from those of Sarwar and Kremer (1992) who reported that isolates from the rhizosphere were more efficient auxin producers than isolates from the bulk soil. The phyllosphere bacterial and fungal isolates from the Western and Coastal regions produced IAA in small percentages (Figure 4e and 4b, f). This is in agreement with earlier report of Lindow and Brandl (2003) who stated that despite of studies focusing on rhizospheric and endophytic plant hormone-synthesizing bacteria, epiphytes are also known to produce such substances. However the phytohormone-mediated roles of bacterial ephyphitic communities on plants are not clear.

## Conclusions

The Mwea, Western and Coastal rice ecosystem harbours diverse microorganisms. Results suggest that some of the indigenous bacteria and fungi were able to fix nitrogen, solubilize phosphates and induce the production of IAA. However the ability to perform these plant growth promoting activities was not the same among the tested bacterial and fungal isolates.

Most of the bacterial isolates from the rhizosphere, rhizoplane as well as the phyllosphere had ARA though at low levels. Bacterial isolates from the rhizosphere and rhizoplane were found to be efficient in P solubilization whereas the fungal isolates were mostly non-solubilizers. Although the percentage of IAA production by the tested isolates was not high, the bacterial isolates performed better than the fungal isolates.

The results therefore suggest that these microorganisms have the potential to be utilized as microbial inoculants to replace chemical fertilizers for sustainable rice cultivation in the Kenyan rice growing regions. Thus the future of biofertilizers based on nitrogen fixing, phosphate solubilizing and IAA producing bacteria and to some extent fungi seems very promising.

## Materials and methods

### Sample collection

Soil and plant samples were collected from paddy fields of rice growing regions in Western, Mwea and Coastal Kenya. Samples were taken from a depth of 0–15 cm in the sampling farms where a zigzag format of sampling was used. The plants were uprooted and separated into the root and shoot systems. The samples were kept separately in paper bags and carried to the laboratory. Samples were kept in a refrigerator at 4°C during the period of experimentation.

### Isolation and screening

#### Isolation of microorganisms

The microbes from phyllosphere, rhizoplane and rhizosphere were isolated on their respective media; bacterial isolates on nutrient agar (NA) and fungal isolates on potato dextrose agar (PDA). Serial dilution technique was performed up to 10^-5^ dilution. The cultures were then incubated for 3 and 5 days at 28°C to observe the bacterial colonies and fungal growth respectively. Individual bacterial colonies and fungal structures were selected based on their morphological characteristics, picked and re-cultured on fresh media for purification to generate pure cultures. The pure cultures were maintained at 4°C.

#### Characterization of bacterial isolates

All the isolates were streaked on NA plates and cultures incubated at 28°C for 3 days. Thereafter the morphological characteristics of the colonies such as colour, form, elevation and margin were recorded. Gram stain differentiates bacterial cells into two major groups; gram-positive organisms which stain blue to purple; and gram-negative organisms which stain pink to red. It also classifies the cells into either rods or cocci. Microscopic observations were performed to investigate the Gram reaction and motility characteristics as per the standard methods (Cappuccino and Sherman 2002).

#### Characterization of fungal isolates

Fungal isolates were incubated at 28°C on PDA plates for 7 days. All the fungal isolates were morphologically identified according to colony colour and texture pigmentation (surface, periphery and reverse). Microscopic observation of spores and spore bearing structures were performed by the use of lactophenol cotton blue mounts and the isolates were identified at Genus level according to illustrations by Barnett and Hunter 1987.

#### Nitrogen fixation ability assay

The nitrogen-free (NFb) semisolid medium composed of 1 g K_2_HPO_4_, 0.2 g MgSO_4_.7H_2_O, 1 g CaCO_3_, 0.2 g NaCl, 5 mg FeSO_4_.7H_2_O, 10 g glucose, 5 mg NaMoO_4_, 2.3 g of agar per liter at pH 7.0 was prepared. Five millitres of the media were placed in 10 ml cultural tubes and the bacterial cultures were inoculated and incubated at 30°C for 48 hours. The headspace of the cultural tube was replaced with 10% C_2_H_2_, and the tube was kept at 30°C for 12 hours. C_2_H_4_ production in the headspace was assayed using a Shimadzu gas chromatograph (GC-9A, Japan). The column temperature was 120°C while the injection/detection temperature was 220°C. A needle and syringe were used to pick 1 ml of the free space in the cultural tubes, which was then injected into the GC machine that gave a chromatograph showing retention time of 1.4–1.5 minutes. An un-inoculated tube of the NFb semi-solid medium was used as a control.

#### Phosphate solubilization assay

The phosphate solubilization ability of the bacterial and fungal isolates was tested by plate assay using National Botanical Research Institute’s phosphate (NBRIP) growth medium (Nautiyal 1999). The medium contained in a litre; 10 g glucose, 5 g Ca_3_(PO_4_)_2_, 5 g MgCl_2_, 0.25 g MgSO_4_, 0.2 g KCl, 0.1 g (NH_4_)_2_SO_4_ and 1.5% agar. The pH of the media was adjusted to 7.0. For clear detection and estimation of the phosphate solublization ability of microorganisms, another set of NBRIP media supplemented with 0.1 g of bromophenol blue was also used. The plates were incubated at 28°C. Formation of visible halo zones around the microbial colonies/structures in plates containing NBRIP media and yellow coloured halos in plates containing bromophenol blue was an indication of the phosphate solubilization ability of the microorganisms. The halo and colony/structure diameters were measured at 7 and 14 days after inoculation. Halo size was calculated by subtracting colony/structure diameter from the total diameter.

#### IAA production assay

Detection of IAA production was done as described by Brick (Brick et al. 2004). The pure bacterial and fungal cultures were inoculated and incubated for 48 hours and 72 hours respectively on their respective liquid media at 30°C and 28°C. Fully grown cultures were centrifuged at 3000 rpm for 30 min. The supernatant (2 ml) was mixed with two drops of orthophosphoric acid and 4 ml of the salkowski reagent (50 ml, 35% of perchloric acid, 1 ml 0.5 m FeCl_3_ solution). Development of pink colour after 0.5–2 hours incubation at room temperature indicates IAA production.
